# CYP-450 Epoxygenase Derived Epoxyeicosatrienoic Acid Contribute To Reversal of Heart Failure in Obesity-Induced Diabetic Cardiomyopathy *via* PGC-1 *α* Activation

**DOI:** 10.4172/2329-6607.1000233

**Published:** 2018-02-08

**Authors:** SP Singh, JA McClung, L Bellner, J Cao, M Waldman, J Schragenheim, M Arad, E Hochhauser, JR Falck, JA Weingarten, SJ Peterson, NG Abraham

**Affiliations:** 1 Departments of Pharmacology and Medicine, New York Medical College, Valhalla, New York, USA; 2 Departments of Medicine, New York Medical College, Valhalla, New York, USA; 3 Chinese PLA General Hospital, Beijing 100853, China; 4 Cardiac Research Laboratory, Felsenstein Medical Research Institute and Sackler School of Medicine, Tel-Aviv University, Israel; 5 Leviev Heart Center, Tel Hashomer and Sackler School of Medicine, Tel Aviv University, Israel; 6 Department of Biochemistry, University of Texas Southwestern Medical Center, Dallas, Texas, USA; 7 Weill Cornell Medicine, New York, USA; 8 New York Presbyterian Brooklyn Methodist Hospital, New York, USA; 9 Joan Edward School of Medicine, West Virginia, USA

**Keywords:** Inflammation, Oxidant stress, Cardiomyopathy, Metabolic syndrome, Hypertension, Myocardial biology

## Abstract

We have previously shown that an Epoxyeicosatrienoic Acid (EET) -agonist has pleiotropic effects and reverses cardiomyopathy by decreasing inflammatory molecules and increasing antioxidant signaling. We hypothesized that administration of an EET agonist would increase Peroxisome proliferator-activated receptor-gamma coactivator (PGC-1α), which controls mitochondrial function and induction of HO-1 and negatively regulates the expression of the proinflammatory adipokines CCN3/NOV in cardiac and pericardial tissues. This pathway would be expected to further improve left ventricular (LV) systolic function as well as increase insulin receptor phosphorylation. Measurement of the effect of an EET agonist on oxygen consumption, fractional shortening, blood glucose levels, thermogenic and mitochondrial signaling proteins was performed. Control obese mice developed signs of metabolic syndrome including insulin resistance, hypertension, inflammation, LV dysfunction, and increased NOV expression in pericardial adipose tissue. EET agonist intervention decreased pericardial adipose tissue expression of NOV, while normalized FS, increased PGC-1α, HO-1 levels, insulin receptor phosphorylation and improved mitochondrial function, theses beneficial effect were reversed by deletion of PGC-1α. These studies demonstrate that an EET agonist increases insulin receptor phosphorylation, mitochondrial and thermogenic gene expression, decreased cardiac and pericardial tissue NOV levels, and ameliorates cardiomyopathy in an obese mouse model of the metabolic syndrome.

## Introduction

Heart failure continues to be a serious health crisis with a current estimate of more than 37 million persons worldwide [1]. Of these, the prevalence of diabetes mellitus is 24% in patients with chronic heart failure and 40% in those hospitalized with debilitating heart failure. Chronic obesity and diabetes are potent mediators of diabetic cardiomyopathy [1,2]. A recently discovered adipokine, nephroblastoma overexpressed CCN3 (NOV/CCN3), is a multifunctional protein of the CCN family which is involved in many pathophysiological processes, including inflammation, interstitial fibrosis and renal tissue damage and repair [3]. NOV also modulates cell proliferation, cell adhesion activities and the subsequent induction of proinflammatory cytokines and chemokines in human cardio metabolic patients. Elevated NOV is attributed to increased obesity, plasma triglycerides and C-reactive protein [4]. Elevated NOV has also recently been associated with obstructive sleep apnea [5], a clinical syndrome that is strongly associated to obesity, insulin resistance and cardio-metabolic disease [5,6]. Abolition of NOV was found to decrease fat mass, improve glucose tolerance, insulin sensitivity, and to decrease proinflammatory cytokines and chemokines in adipose tissues of obese mice [7].

Impairment of mitochondrial energetics, increased reactive oxygen species (ROS) production and the resultant oxidative stress induces hypertension [8,9], and are considered primary risk factors in the development of diabetic cardiomyopathy [10]. Mitochondria play a major role in ROS generation and maintain an intricate balance between fusion and fission events [10]. The formation of interconnected mitochondrial networks via fusion allows for the replication and distribution of mtDNA across the reticulum, and is mediated by dynamin GTPases, mitofusin (Mfn1 and Mfn2 [10,11]. In contrast, the fission-induced dispersion of mitochondrial networks is governed by dynamin-related protein 1 (DRP1) and mitochondrial fission 1 (Fis1).

Obesity and ROS induction contributes to a reduction in HO-1 levels by either inherited or sedentary lifestyle-induced dyslipidemia, a decline in respiratory capacity, OXPHOS machinery, and ATP production [12–14], contributing to mitochondrial dysfunction and reduced energy expenditure [15–17]. Moreover, oxidative mutations of mitochondrial fusion and fission proteins disable the capability of mitochondrial dynamics to maintain mtDNA integrity, further enhancing ROS-mediated oxidative stress [18,19].

A master regulator of mitochondrial biogenesis is peroxisome proliferator-activated receptor gamma coactivator-1 alpha (PGC-1α), which regulates respiratory chain complexes and ATP synthases [20–22]. PGC-1α levels in cardiomyocytes are critical to mitochondrial polarization, adequate output of ATP for cardiac energy supply, and increases in VO_2_ [22,23]. As excessive adipose tissue accumulation is a significant source of ROS and pro-inflammatory cytokines that may ultimately result in LV diastolic dysfunction, increased fibrosis and decreased contractility [14,24,25]. Ectopic fat accumulation and inflammation adjacent to the epicardium and pericardium has local deleterious effects on cardiac structure and function, further contributing to cardiomyopathy [24,26–28]. We previously showed that PGC-1α is regulated by EETs in adipocyte cells [29]. EETs are generated by a family of cytochrome P450 (CYP) monooxygenases and epoxygenases through their metabolism of arachidonic acid [30,31]. While ROS can rapidly hydrolyze EETs (reviewed in [14], EET agonists prevent both vascular complications and adiposity, while expansion of adipose tissue impairs the generation of EETs *in vivo* [32–35].

We hypothesize that the beneficial effect of EET-agonists on cardiac function is mediated by a reduction in the local secretion of inflammatory molecules such as NOV by pericardial fat, accompanied by upregulation of PGC-1α and HO-1 and amelioration of obesity-mediated cardiomyopathy. We further hypothesize that EET upregulation may change the pericardial adipocyte phenotype from white to beige-like adipocytes resulting in increased expression of thermogenic genes and improved mitochondrial biogenesis and function.

## Materials and Methods

### Cell culture

#### Generation of PGC-1α-deficient cells using lentivirus

3T3-L1 murine pre-adipocytes were purchased from ATCC (ATCC, Manassas, VA). Cells were cultured in α-minimal essential medium (α-MEM, Invitrogen, Carlsbad CA) supplemented with 10% fetal bovine serum (FBS, Invitrogen, Carlsbad, CA) and 1% antibiotic/antimycotic solution (Invitrogen, Carlsbad, CA). The cultures were maintained at 37°C in a 5% CO2 incubator and the medium was changed after 48 h and every 3~4 days thereafter, as described previously [29]. SMART vector lentiviral shRNA-PPARGC1A or scrambled RNA (Dharmacon, Lafayette, CO) was applied to 3T3-L1 cells to establish a stably transduced cell line, as described [29].

### Animal protocols

We used db/db mice as a model of obesity induced diabetic cardiomyopathy as these mice develop insulin resistance, hyperglycemia and fail to control gluconeogenesis, leading to peripheral neuropathy and myocardial disease. Sixteen week old male db/db mice, on C57BL/6J background (Jackson Labs, Bar Harbor, ME), were divided into four treatment groups, 6 mice per group, following a 16-week acclimatization period weighing approximately 54 g at the start of the experiment (data not shown):

- Control
- Ln-non-target-shRNA
- EET-agonist
- EET-agonist plus Ln-PGC-1α-shRNA

Age-matched C57BL/6J mice (C57) (Jackson Labs) were used as WT (lean, C57) controls. All mice were fed regular (Harlan, Teklad Lab Animal Diets, US). Mice (db/db) were treated as follows:

- Control
- Administered 2 bolus injections of 40–70 × 10^6^ TU/mouse in 80–100 µl of Ln-non-target-shRNA (Dharmacon, Lafayette, CO, US) into the retro orbital vein,
- Injected intraperitoneally with the EET-agonist twice/week for eight weeks at a dose of 15 mg/kg body weight,
- EET-agonist and administered 2 bolus injections 40–70 × 10^6^ TU/mouse in 80–100 µl PGC-1α (sh) lentivirus (Dharmacon) into the retro orbital vein at 3 and 5 weeks after the start of EET-agonist injections.

At the end of the study, mice were euthanized with ketamine (100 mg/kg)/xylazine (10 mg/kg) administration followed by cervical dislocation. Body weight and fat content were measured at sacrifice. All animal studies have been approved by the appropriate ethics committees of the NYMC, IACUC institutionally approved protocol in accordance with the NIH guidelines.

### Fasting blood glucose, glucose tolerance test and blood pressure measurement

Fasting blood glucose measured following a 6 h fast. For the glucose tolerance test, mice were injected intraperitoneally with glucose (2.0 g/kg body weight) after which blood glucose was measured every 30 minutes up to 120 min. Blood pressure measured using standard tail-cuff method [36].

### Echocardiogram measurement of fractional shortening

Echocardiography was performed using a 12-MHz probe on isoflurane anesthetized mice. Images of the left ventricular (LV) diameter was obtained in M-mode and used to measure the end-diastolic and end-systolic diameters from which the LV fractional shortening was calculated [36].

### Determination of oxygen consumption and respiratory quotient

The Oxylet gas analyzer and air flow unit (Oxylet; Panlab-Bioseb, Vitrolles, France) was used to determine individual mouse VO_2_ and RQ as previously described [36].

### ATP and mitochondrial copy number measurement

ATP content was quantified in heart tissue (10 mg/mouse) using an ATP Colorimetric Assay Kit (ABCAM, Cambridge, MA) as per the instructions provided by the manufacturer. Mouse Mitochondrial DNA Copy Number Kit (Detroit R&D, Inc., Detroit, MI) was used to measure mtDNA Copy Number.

### Real-time qPCR and western blot analysis

For real-time PCR analysis total RNA was extracted from heart and pericardial fat tissues with TRIzol^®^ (Ambion, Austin, TX). A High Capacity cDNA Reverse Transcription Kit (Applied Biosystems) was used to synthesize cDNA from total RNA. Specific TaqMan^®^ Gene Expression Assay probes (Fisher Scientific, Waltham, MA) for mouse PGC-1α, HO-1, NOV/CCN3, COX-1, PRDM16, CCL2, Adiponectin, TNFα, IL-6 and GAPDH were used as previously described. For Western blot, tissues were first lysed in RIPA buffer supplemented with protease and phosphatase inhibitors (Complete™ Mini and PhosSTOP™, Roche Diagnostics, Indianapolis, IA) and total protein content was analyzed by the Bradford method (BIO-RAD, Hercules, CA). Immunoblotting was performed for select proteins [18,36].

### Measurement of HO and EET levels

Heart tissue was lysed in RIPA lysis buffer, CO released and EET levels were analyzed as previously described [18,36,37].

### Statistical analysis

Data values are expressed as means ± S.E.M. Student’s t-test was used for pairwise comparison and one-way ANOVA with Bonferroni’s post-test for comparison was used to calculate the significance of mean value differences using one-way analysis of variance. The null hypothesis was rejected at p<0.05.

## Results

### EET-agonist administration increases endogenous EET levels and improves metabolic function

Although the db/db mice treated with the EET agonist ate the same amount of food as the other groups during treatment, they weighed significantly less at the experimental endpoint 45.2 ± 1.33 g, than control mice (p<0.05) (63.9 ± 1.58 g). Knockdown of PGC-1α reversed the beneficial effect of EET-agonist with mice weighing 54.9 ± 3.65 g at the experimental endpoint (Table 1). Fasting blood glucose in the db/db control, and EET-agonist and EET-agonist with PGC-1α (sh) treated mice was 355 ± 22.2 mg/dL, 134 ± 6.5 mg/dL, and 205 ± 5.5 mg/dL, respectively (Table 1). Knockdown of PGC-1α reversed the effects of EET-agonist treatment. As seen in Table 1, EET-A treatment of db/db mice improved (p<0.05) glucose tolerance as compared to db/db control, an effect that was inhibited in ln-PGC-1α (sh) db/db mice.

Blood pressure was reduced (p<0.05), oxygen consumption increased (p<0.05), and the CO_2_/O_2_ ratio was decreased with EET-agonist treatment (p<0.05) (Table 1). These EET-agonist-mediated effects were significantly reduced in EET-agonist-treated mice administered Ln-PGC-1α (sh). Obesity was associated with an increase in heart weight as compared to mice treated with the EET-agonist alone (0.16 ± 0.004 *vs.* 0.13 ± 0.001, respectively), while the EET-agonist in combination with decreased levels of PGC-1α prevented the beneficial effect of EET-agonist on heart weight (0.14 ± 0.006) (Table 1).

Importantly, EET-agonist is known to inhibit sEH, and therefore may directly result in the observed elevation of endogenous cellular levels of EET in cardiac tissues, an effect that was prevented in PGC-1α-deficient mice treated with EET-agonist (Table 1).

Glucose tolerance was improved (p<0.05) in db/db mice treated with EET-agonist with a decrease in fasting glucose, suggesting increased insulin sensitivity (p<0.05) (Table 1). This was partially prevented (p<0.05) in PGC-1α-deficient db/db mice (Table 1).

### EET-agonist treatment restores cardiac function and associated signaling pathways

**Figure 1:**
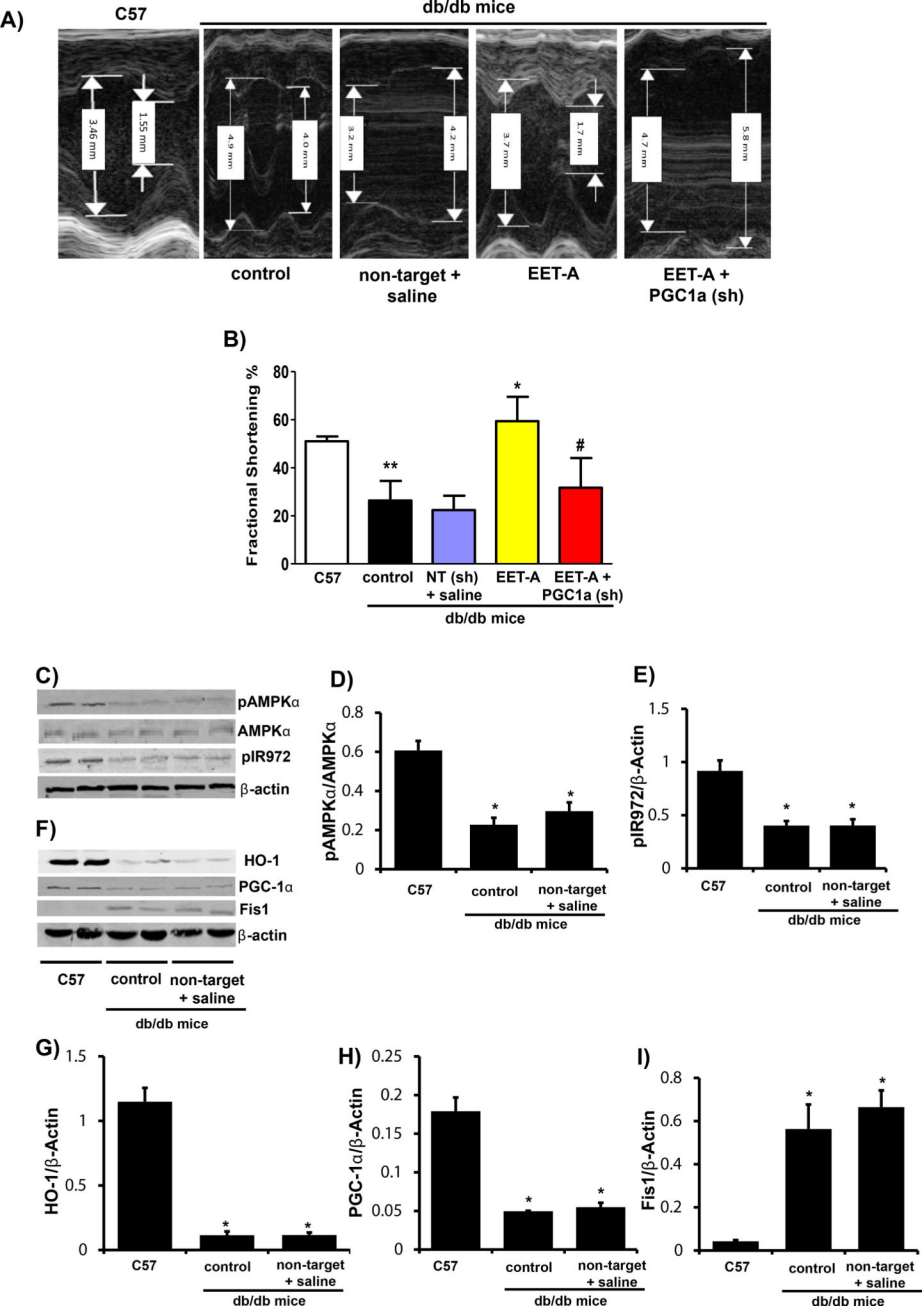
EET-agonist treatment improves left ventricular dilation, fractional shortening, and glucose tolerance. **(A)** Sample M-mode short axis echocardiographic images showing LV dilation and FS in wild-type (C57) and db/db control, db/db non-target and vehicle (saline) injected, db/db treated with EET-agonist, and PGC-1α-deficient db/db mice treated with EET-agonist; **(B)** Graph depicting left ventricular fractional shortening in each cohort (n=6, **p<0.05 *vs.* C57, *p<0.05 *vs.* control and ^#^p<0.05 *vs.* EET-agonist). Results are mean ± SE, n=6, *p<0.05 *vs.* db/db control, #p<0.05 *vs.* db/db mice treated with EET-agonist. Representative blots; **(C)** and densitometric analysis of **(D)** pAMPKα/AMPKα, and **(E)** pIR972, representative blots **(F)** and densitometric analysis of **(G)** HO-1, **(H)** PGC-1α, and **(I)** Fis1 in WT (C57), db/db control and db/db non-target and vehicle (saline) injected mice. Results are mean ± SE, n=6, *p<0.05 *vs.* C57 mice.

Cardiac function, as measured by echocardiography and calculation of fractional shortening (FS), was significantly impaired (p<0.05) in control db/db mice as compared to lean WT (C57) (Figure 1A and B). Fractional shortening was improved (p<0.05) in db/db mice treated with EET-agonist (p<0.05) (Figure 1A and B), an effect that was partially prevented (p<0.05) in PGC-1α-deficient db/db mice (Figure 1A and B). Injection of db/db mice with saline and non-target lentiviral vector (vehicle and vector controls) had no effect on FS as compared to control db/db mice (Figure 1A and B).

Cardiac tissues from control db/db mice expressed lower levels (p<0.05) of pAMPKα and pIR972 as compared to C57 (WT C57Bl6) mice (Figure 1C–E). Decreased phosphorylation of AMPKα in db/db mice was associated with a reduction in phosphorylation of the insulin receptor (Figure 1E). HO-1 and PGC-1α protein levels in db/db mice were lower (p<0.05) compared to C57 mice (Figure 1F–H). Decreased levels of HO-1 and PGC-1α in db/db mice were associated with an increase in Fis, indicative of increased mitochondrial fission (Figure 1I). As seen in Figure 1 treatment of db/db with saline and non-target lentiviral vector (vehicle and vector controls) had no effect on any of the protein levels as compared to control db/db mice.

### EET-agonist administration causes reduction of NOV expression

**Figure 2:**
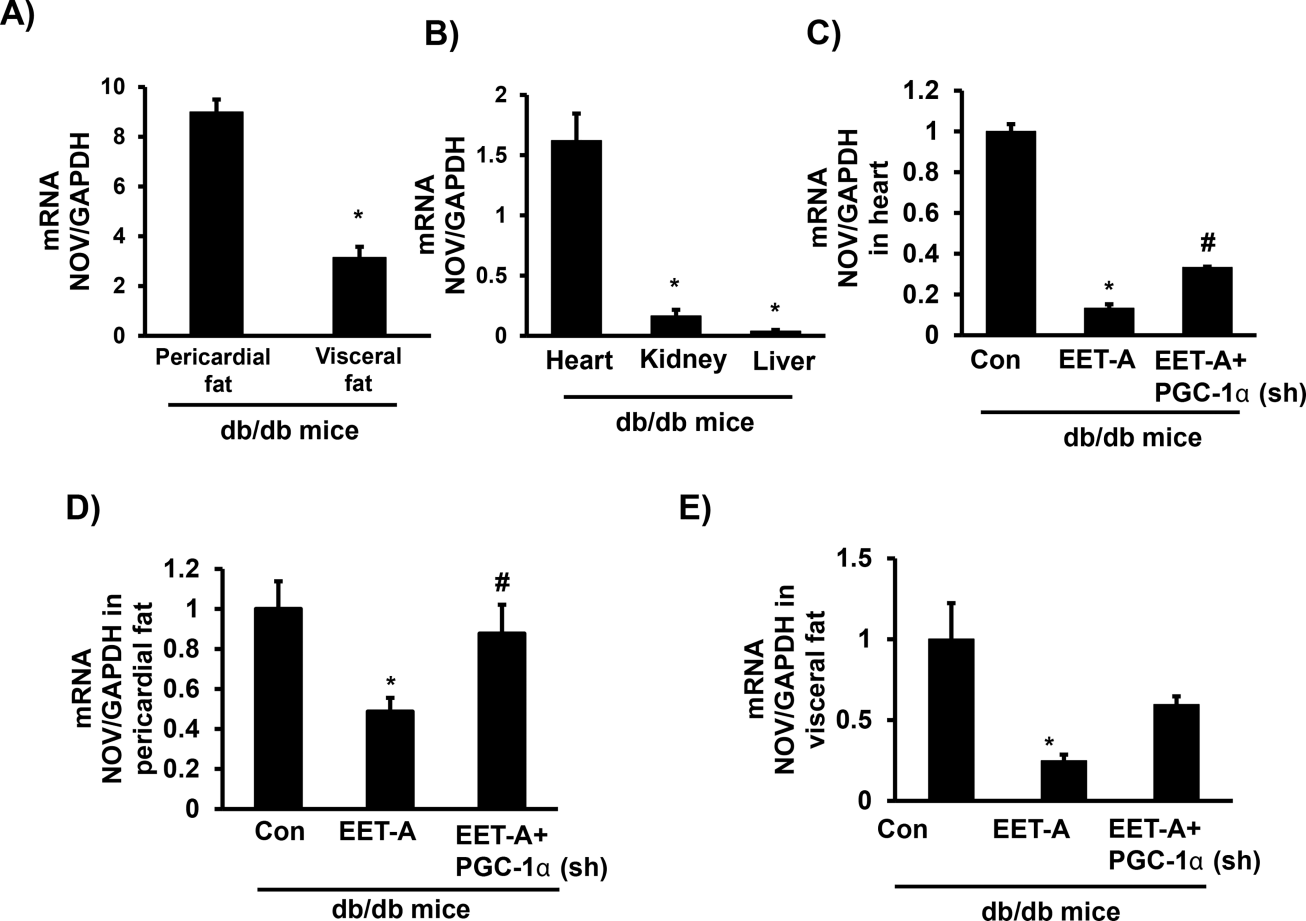
Expression of NOV in different tissues of control db/db mice. **(A)** NOV mRNA expression in pericardial fat and visceral fat; **(B)** NOV mRNA expression in heart, kidney and liver; **(C)** Effect of EET-agonist treatment on NOV mRNA expression levels in heart of individual mouse, Effect of EET-agonist treatment on NOV mRNA expression in **(D)** Pericardial fat and **(E)** Visceral fat of db/db control mice, db/db treated with EET-agonist mice, and PGC-1α-deficient db/db mice treated with EET-agonist. Results are mean ± SE, n=6, *p<0.05 *vs.* db/db control, ^#^p<0.05 *vs.* db/db mice treated with EET-agonist.

Basal levels of pericardial adipose tissue expression of NOV were significantly higher than in visceral fat (p<0.05) (Figure 2A). Cardiac tissues expressed the highest levels of NOV compared to kidney and liver (p<0.05) (Figure 2B). EET-agonist administration decreased NOV in cardiac and pericardial adipose tissues (Figure 2C and D). Importantly, a reduction in PGC-1α reversed the ability of EET-agonist to reduce NOV in both cardiac and pericardial adipose tissues (Figure 2C and D). A similar trend was observed in visceral adipose tissue (Figure 2E).

### PGC-1α knockdown prevented EET-agonist-mediated increase in Sirt1, phosphorylation of AMPKα, and IR972 in cardiac tissue

**Figure 3:**
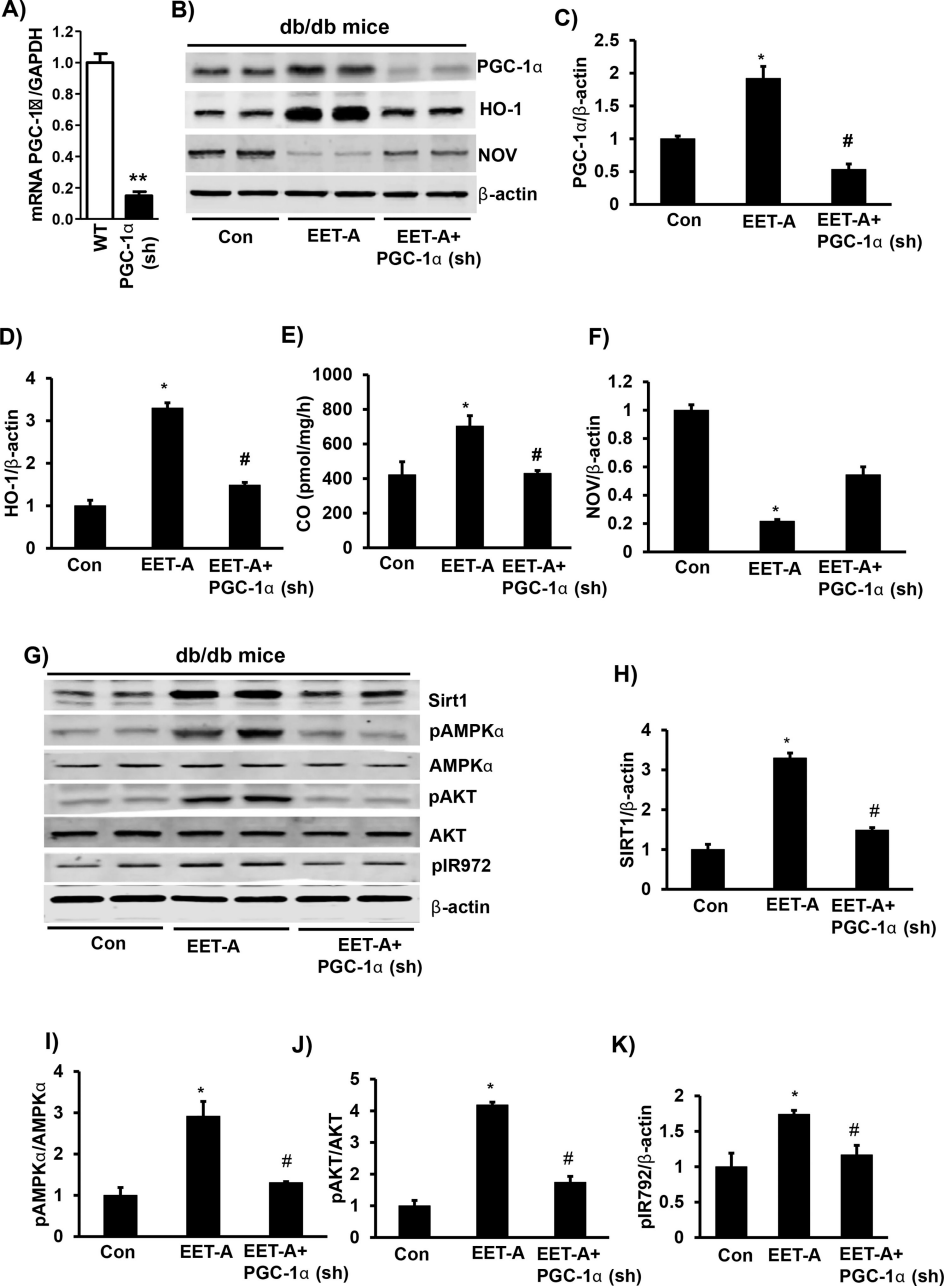
Effect of EET-agonist treatment on levels of PGC-1α, HO-1, NOV Sirt1, pAMPKα, total AMPKα, pAKT, total AKT and pIR972 in cardiac tissue of db/db mice. **(A)** PGC-1α mRNA levels in control (WT) and lentivirus-mediated knockdown of PGC-1α in 3T3-L1-derived adipocytes. Representative western blots; **(B)** and **G**) and densitometric analysis of **(C)** PGC-1α, **(D)** HO-1, **(E)** HO-activity as measured by CO generation, **(F)** NOV, **(H)** Sirt1, **(I)** pAMPKα/AMPKα, **(J)** pAKT/AKT, and **(K)** pIR972 in cardiac tissues of db/db control mice, db/db mice treated with EET-agonist, and PGC-1α-deficient db/db mice treated with EET-agonist. Results are mean ± SE, n=6, *p<0.05 *vs.* db/db control, ^#^p<0.05 *vs.* db/db mice treated with EET-agonist.

Figure 3A shows efficient lentivirus-mediated knockdown of PGC-1α in 3T3-L1 derived adipocytes *in vitro.* EET-agonist-mediated induction of PGC-1α, HO-1 expression and HO-activity, was associated with a decrease in NOV expression (Figure 3B–F). EET-agonist led to a 2-fold upregulation of PGC-1α in cardiac tissues compared to control mice (Figure 3B and C). The EET- agonist-mediated increase in PGC-1α levels was prevented in Ln-PGC-1α (sh) mice (Figure 3B and C). HO-1 levels were increased in EET-agonist treated mice compared to control mice (p<0.05) (Figure 3B and D). Ln-PGC-1α (sh) in EET-treated mice prevented the EET-agonist-mediated induction of HO-1 expression and reduced it to the level of db/db control mice (Figure 3B and D). The effect of EET-agonist and PGC-1α knockdown on HO-1 protein levels was paralleled by measurement of HO-activity (Figure 3E). Importantly, NOV protein levels were reduced (p<0.05) (Figure 3F) in cardiac tissue of EET-agonist treated mice, an effect reversed in PGC-1α-deficient mice, confirming the changes observed in mRNA levels (Figure 2). EET-agonist treatment increased Sirt1 and phosphorylation of both AMPKα (Thr172) and AKT (Ser473) levels (p<0.05) mediating an increase in insulin receptor phosphorylation and prevented by lentivirus-mediated silencing of PGC-1α (Figure 3G–K).

### Increased EET-levels in cardiac tissues improves mitochondrial function and biogenesis

**Figure 4:**
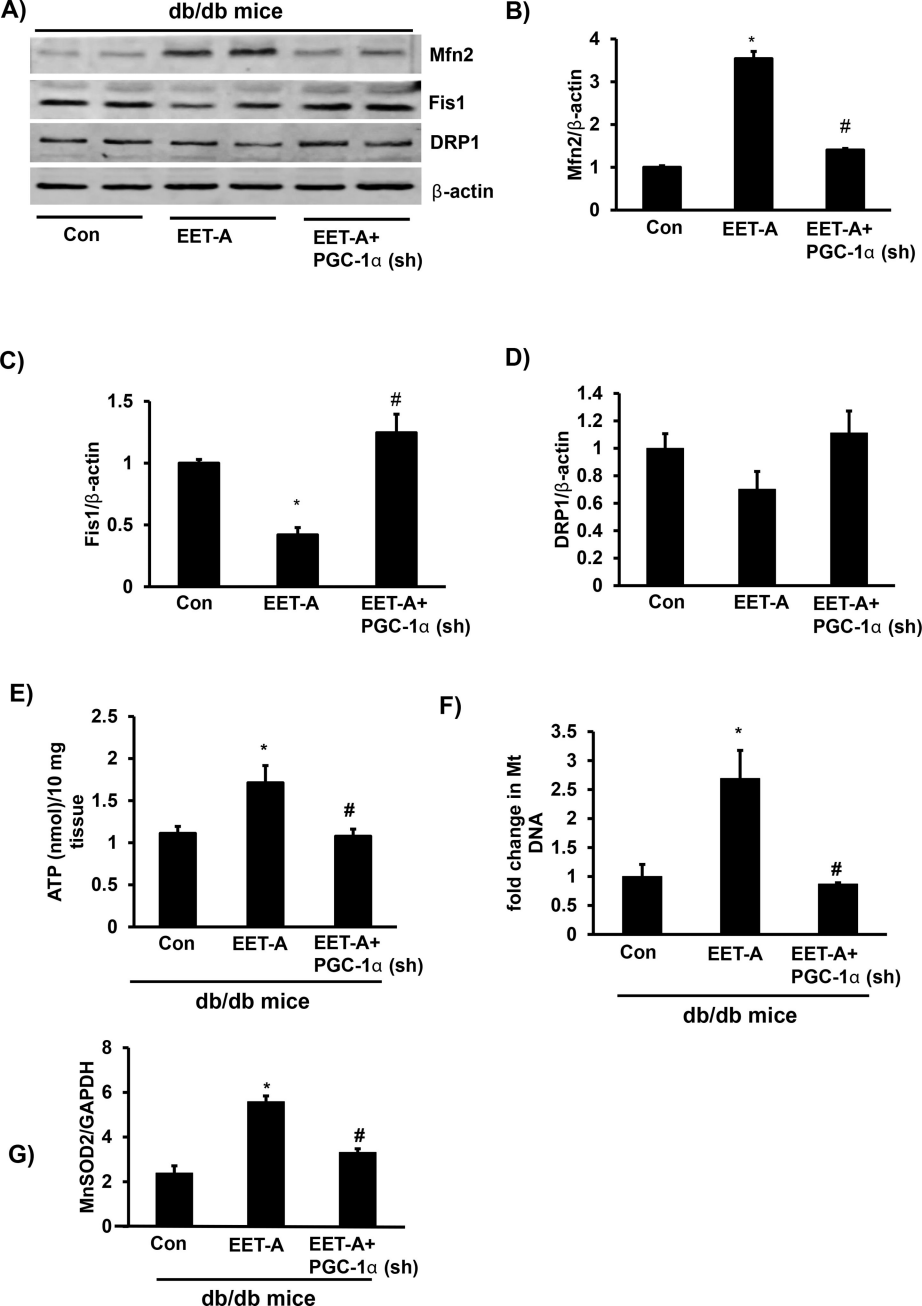
Effect of EET-agonist treatment on mitochondrial function and biogenesis in cardiac tissue of db/db mice. **(A)** Representative western blots, densitometric analysis of **(B)** Mfn2; **(C)** Fis1, and **(D)** DRP1, **(E)** ATP levels, **(F)** mitochondrial copy number; **(G)** mRNA expression of an antioxidant gene MnSOD2 in cardiac tissues of db/db control mice, db/db mice treated with EET-agonist, and PGC-1α-deficient db/db mice treated with EET-agonist. Results are mean ± SE, n=6, *p<0.05 *vs.* db/db control, #p<0.05 *vs.* db/db mice treated with EET-agonist.

EET-agonist increased Mfn2 expression and decreased Fis1 (Figure 4A–C). The EET-agonist-mediated effect on Mfn2 and Fis1 was reversed in PGC-1α-deficient mice (Figure 4A–C). The levels of DRP1 were not impacted by either EET-agonist or lentivirus-mediated suppression of PGC-1α (Figure 4A and D). EET-agonist improved both mitochondrial function and biogenesis in cardiac tissues of EET-agonist treated mice as assessed by the increase in ATP generation and mitochondrial copy number. In contrast, in cardiac tissues of PGC-1α-deficient mice treated with EET-agonist both ATP generation and mitochondria copy number were significantly (p<0.05) decreased (Figure 4E and F). Furthermore, EET-agonist increased mRNA expression levels of an antioxidant gene MnSOD2 in cardiac tissue of mice, the beneficial effects of EET was abolished by lentivirus-mediated suppression of PGC-1α (Figure 4G).

### EET-agonist treatment reduces inflammatory cytokines levels and cardiac remodeling (MMP2)

**Figure 5:**
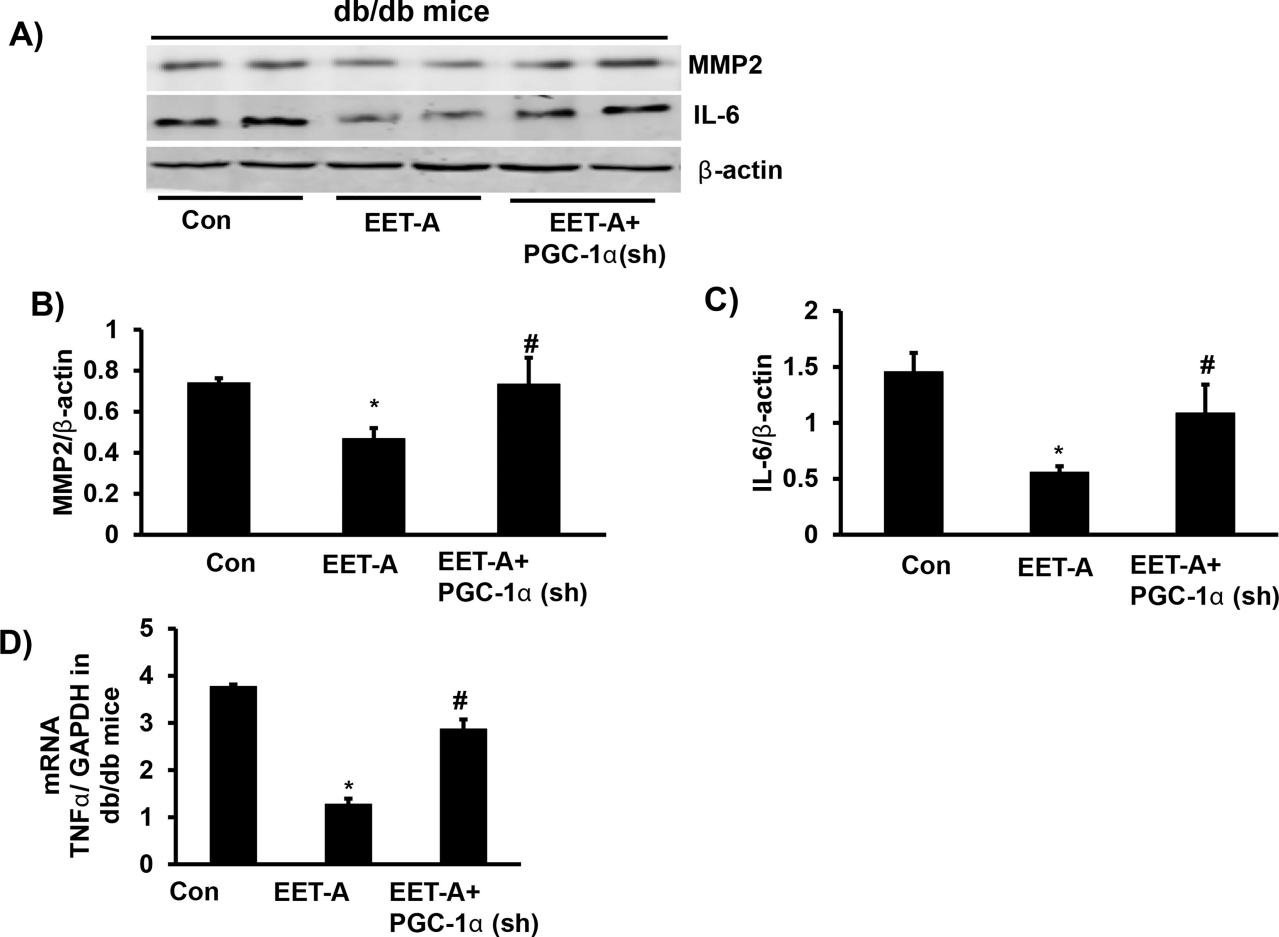
Effect of EET-agonist treatment on inflammatory cytokine and MMP2 levels in cardiac tissue of db/db mice. Representative blots **(A)** and densitometric analysis of **(B)** MMP2, **(C)** IL-6, and **(D)** mRNA expression of TNF-α in cardiac tissue of db/db control mice, db/db mice treated with EET-agonist, and PGC-1α-deficient db/db mice treated with EET-agonist. Results are mean ± SE, n=6, *p<0.05 *vs.* db/db control, ^#^p<0.05 *vs.* db/db mice treated with EET-agonist.

The cardiac expression of MMP-2 protein was reduced in EET-agonist treated db/db mice compared to control db/db mice (p<0.05), which was prevented by Ln-PGC-1α (sh) (Figure 5A and B). EET-agonist-mediated decrease of MMP-2 may explain the prevention of cardiac remodeling, an effect reversed by silencing of PGC-1α (Figure 5A–D). TNFα and IL-6 in cardiac tissues of EET-agonist treated db/db mice were reduced compared to cardiac tissues of control db/db mice (Figure 5A–D), a result similar to pericardial fat tissue.

### EET-agonist administration increases thermogenic and mitochondrial genes in pericardial adipose tissues

**Figure 6:**
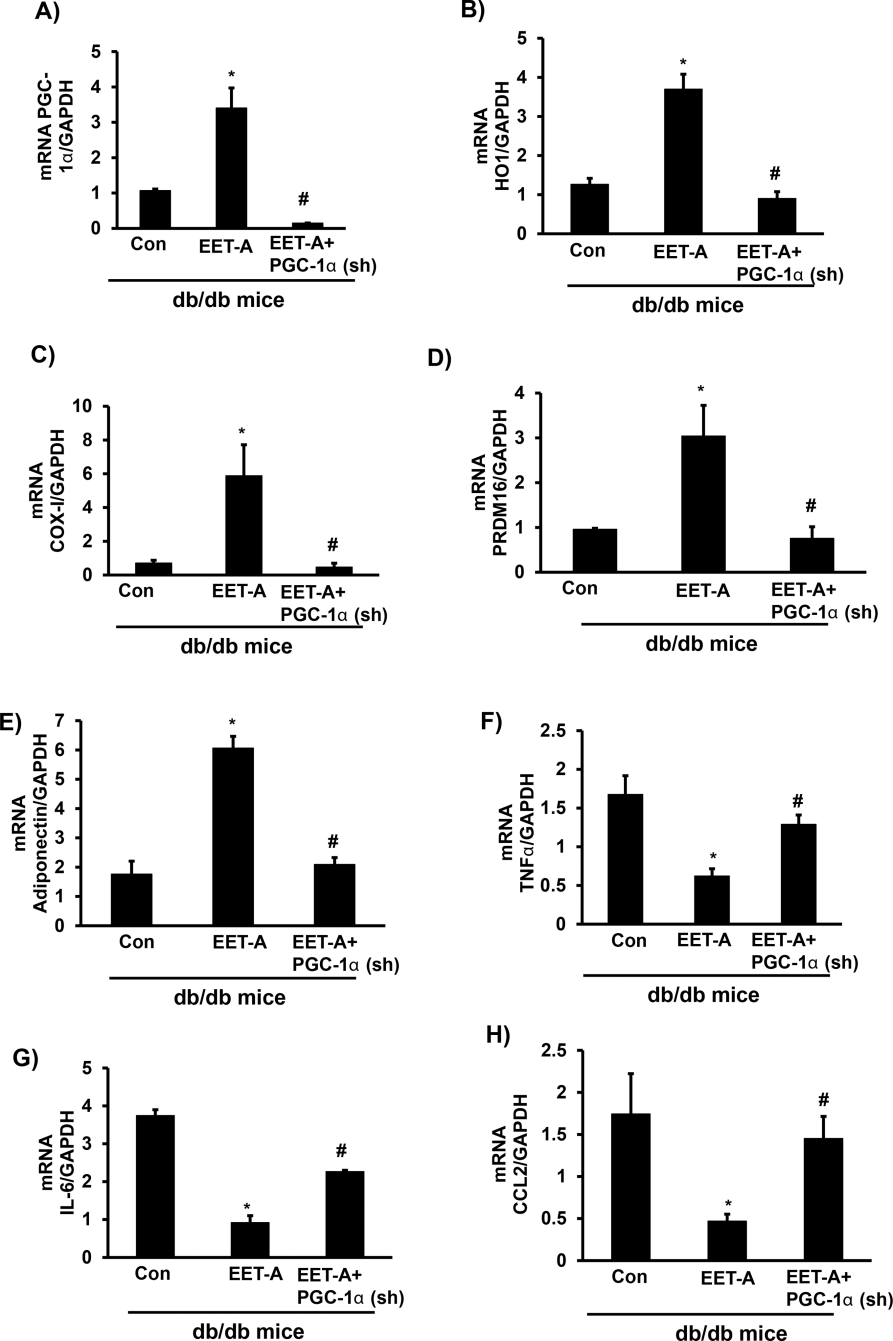
Effect of EET-agonist treatment on levels of PGC-1α, HO-1, COX-I and PRDM16, adiponectin, TNF-α, IL-6, and CCL2 in pericardial adipose tissues of db/db mice. mRNA levels of **(A)** PGC-1α, **(B)** HO-1, **(C)** COX-I, **(D)** PRDM16, **(E)** Adiponectin, **(F)** TNF-α, **(G)** IL-6, and **(H)** CCL2. Results are mean ± SE, n=6, *p<0.05 *vs.* db/db control, ^#^p<0.05 *vs.* db/db mice treated with EET-agonist.

The effect of EET-agonist on cardiac tissue extended to pericardial adipose tissue. This is evidenced by increased levels of HO-1, PGC-1α, and PRDM16 (thermogenic genes), and increased mitochondrial function (COX-I) in pericardial adipose tissue (Figure 6A–D). EET-agonist treated db/db mice exhibited increased PGC-1α mRNA levels compared to db/db control mice (p<0.05), whereas PGC-1α mRNA expression in pericardial adipose tissue of EET-agonist-PGC-1α (sh) db/db mice was, as expected, lower than control db/db mice (p<0.05; Figure 6A). EET-agonist increased HO-1 mRNA levels compared to control mice (p<0.05), that was prevented in Ln-PGC-1α (sh) mice (Figure 6B). COX-I mRNA levels increased in EET-agonist treated mice (p<0.05) compared to control mice, that was prevented in Ln-PGC-1α (sh) mice (Figure 6C). PRDM16 is a thermogenic gene responsible for the conversion of white fat to brown fat. EET-agonist treated mice expressed higher PRDM16 mRNA levels as compared to control mice (p<0.05), while lentiviral-mediated suppression of PGC-1α reversed the effect of EET-agonist treatment (Figure 6D). Similarly, there was an increase in adiponectin levels and decrease in TNF-α, IL-6 and the monocyte chemoattractant CCL2 (Figure 6E–H). Adiponectin mRNA levels were upregulated in mice administered EET-agonist as compared to control mice (p<0.05), an effect that was blocked in PGC-1α-deficient mice (Figure 6E). EET-agonist treated db/db mice exhibited a decrease in TNF-α, IL-6 and CCL2 mRNA expression levels in pericardial adipose tissue as compared to control mice (p<0.05), while lentivirus-mediated silencing of PGC-1α prevented the EET-agonist-mediated effect (Figure 6 F–H).

## Discussion

This report demonstrates that an EET agonist increases PGC-1α-HO-1, associated with increased insulin receptor phosphorylation, a decrease in fasting blood glucose and a reduction in pericardial fat deposition while decreases cardiac and pericardial fat NOV expression. Pericardial adipose tissue is a metabolically active organ that secretes several adipokines including NOV that may be linked to LV dysfunction. EET mediated decrease in NOV and the concurrent increase in PGC1a-HO-1 results in an increase in thermogenic genes and improved mitochondrial biogenesis and function. Importantly, this suggests that EET-mediated decreases in local expression of NOV in pericardial fat may have a direct effect on LV fractional shortening.

EET-agonist treatment produced an improvement in left ventricular FS, an effect that was reversed in mice with reduced levels of PGC-1α and increased levels of NOV. These results are in accordance with previous observations that PGC-1α deficiency caused LV dilation and poor cardiac contractility [38], insulin resistance and increased circulating lipid levels [39]. Our results suggest a new function for EET that is associated with a decrease in NOV levels. Additionally, we demonstrate that EET-mediated suppression of pericardial fat secretion of NOV accompanied by increased expression of PGC-1α –HO-1 and adiponectin. Thus, there seems to be an opposing relationship between NOV and adiponectin, both in terms of expression and effect on adipocyte maturation and function. Increased NOV in obesity may potentiate adipocyte inflammation and hyperplasia that in turn further increases inflammatory adipokines. EET-mediated decrease of NOV and increase in PGC-1α-HO-1 expression results in improved metabolic parameters in db/db mice highlighting that NOV, PGC-1α and HO-1 are inter-related and have a major relationship in the development of cardiomyopathy. NOV regulates various inflammatory molecules, which impair insulin signaling and promote insulin resistance. Increased NOV levels are associated with obesity, fat deposition and insulin resistance in human cardio metabolic patients [4]. Our results are supported by the observation that low levels of NOV are associated with increased PGC-1α expression, decreased fat mass, increased insulin sensitivity and decreased inflammation in adipose tissue of mice fed a HFD [7]. However, the reciprocal relationship between PGC-1α and NOV in pericardial fat and the high levels of NOV in pericardial adipose tissue compared to the heart, suggest that the regulation of NOV in pericardial adipose tissue is different from that in cardiac tissue.

We demonstrate that EET mediated increases in PGC-1α results in an increase in Mfn2, cytochrome C oxidase and ATP levels, which identify PGC-1α as a major regulator of mitochondrial function [18,36]. Since HO-1 enhances mitochondrial integrity and function and EET-agonist treated mice deficient in PGC-1α display diminished levels of HO-1, reduced levels of Mfn2, ATP, COX-I, and increased levels of Fis1 make it is likely that EET mediates signaling through the PGC-1α and HO-1 pathway. This also confirms that PGC-1α is upstream of HO-1, and is therefore primarily responsible for the HO-1 mediated decrease of pro-oxidants including excess heme seen *in vitro* and in animal models of obesity [34,35]. Heme abatement results in decreased levels of ROS and oxidative stress, both known causes of mitochondrial fragmentation and cardiomyopathy [12,40]. Reduction of HO-1 expression increases heme-mediated ROS levels, resulting in an increase in inflammation [40,41]. HO-1 deficiency causes organ damage in humans and mice [42]. Importantly, gene polymorphisms that negatively regulate the global as well as monocyte-specific HO-1 expression has been shown to be associated with the risk of CVD and atherosclerosis [43,44]. Further, ablation of HO-1 in adipose-specific HO-1 knockout increases ROS through inhibition of mitochondrial fusion and PGC-1α [18]. HO-1 degrades heme to ferrous iron, biliverdin and CO and recent studies indicate that increased HO activity, bilirubin and CO production reduces oxidative stress, and pathological remodeling of the heart [25,45]. HO-1 and CO provides cardioprotection [46], reduces infarct size [47], and protects human stem cells against cell death [48]. The protective effects of increased HO activity also prevent mitochondrial DNA depletion and improved mitochondrial quality control [34,40]. HO-activity plays an important role in mitochondrial electron transport, biogenesis and viability [12,49]. The observation that EET-agonist increases mitochondrial fusion and decreases mitochondrial fission supports the contention that HO activity is mediated by PGC-1α, and is consistent with prior observations that increased HO-1 levels, as well as CO-releasing molecules, exert cardio-protective, anti-fibrotic, and anti-apoptotic effects in both ischemic and non-ischemic cardiomyopathy [46,50].

A relationship between pericardial fat and cardiac contractility is supported by the observation that EET-agonist reduced pericardial adipose tissue deposition, NOV, and TNF-α, IL-6 and CCL2 levels. EETs possess the ability to reprogram pericardial fat, altering phenotype from inflammatory white to healthy functional beige adipocytes with increased adiponectin levels and normal mitochondrial function that express thermogenic genes. These include PRDM16, responsible for the attenuation of cardiac remodeling and cardiomyopathy and associated with increased energy expenditure in, and browning of, adipose tissue, leading to synthesis of adipokines and obesity [51]. Ectopic fat accumulation within and around the heart increases risk of cardiomyopathy associated with mitochondrial dysfunction, highlighting the complex relationship between obesity, insulin resistance and cardiac LV abnormalities [24,26–28,52]. Pericardial and epicardial adipose tissues are a major source of adipokines, inflammatory cytokines and ROS, which can contribute to cardiac remodeling [53].

**Figure 7:**
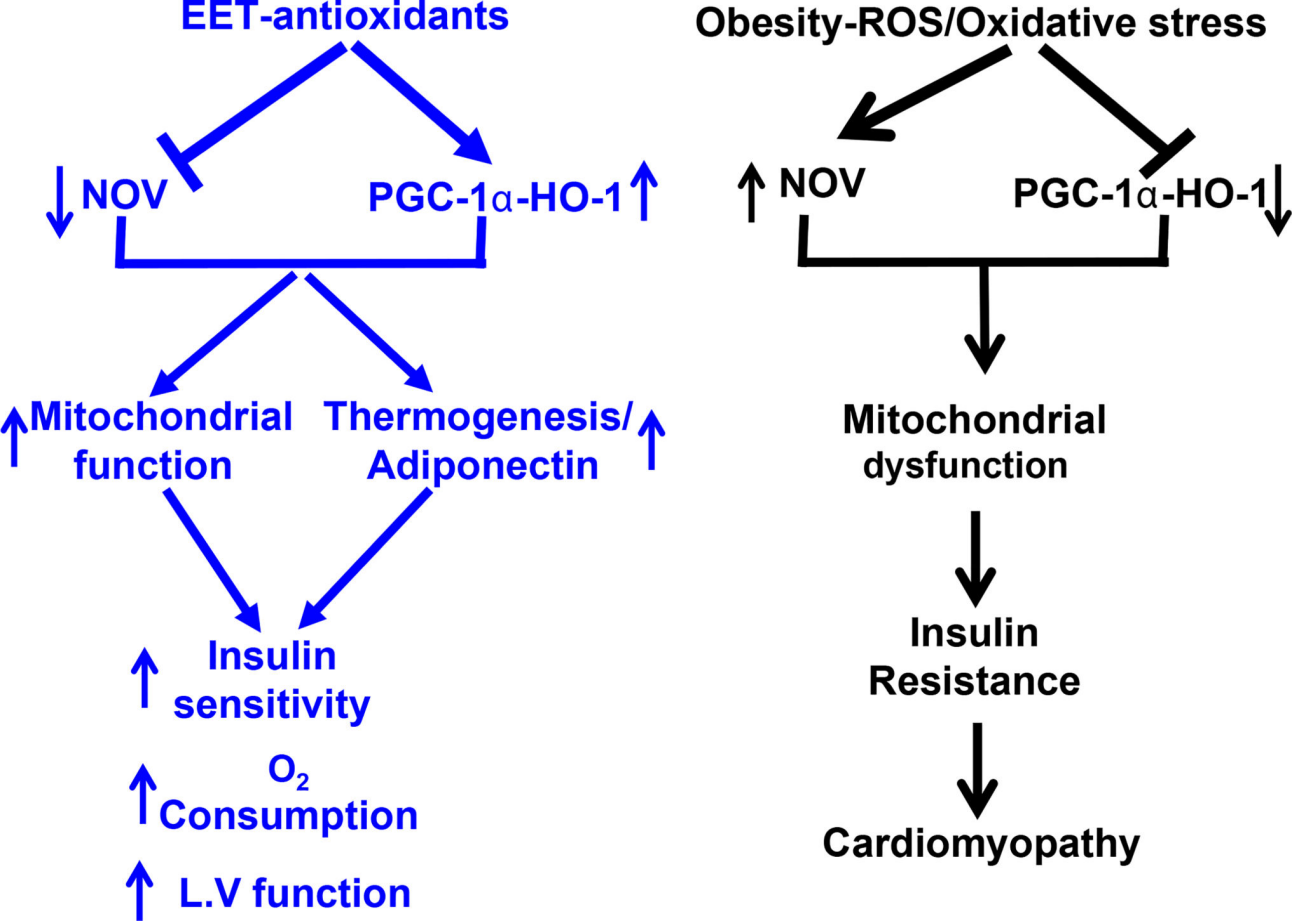
Schematic description of the EET mediated NOV-PGC-1α-HO-1 in mitochondrial biogenesis and function, and thermogenic genes PRDM16. EET-mediated change of pericardial fat phenotype and increase in to expression of mitochondrial signaling protein, thermogenic genes similar to that described to beige type like adipose tissue. The EET-agonist-mediated reduction in NOV subsequently increases PGC-1α and HO-1 leads to increased ATP production, oxygen consumption, increased mitochondrial fusion, DNA copy number, insulin sensitivity that all in concert leads to an improvement in cardiac function.

EET-induced PGC-1α activation reduced the levels of MMP-2 in cardiac tissue, providing mechanistic support for prior observations that EET decreases cardiac fibrosis [25]. EET-related decrease in MMP-2 and improvement in FS is accompanied by activation of AKT and AMPK in cardiac tissue. Likewise, in metabolic organs such as the liver, adipose tissue, and skeletal muscle, activation of AMPK stimulates catabolic and inhibit anabolic processes [54]. EET-agonists offer a unique multifactorial clinical approach to prevention and treatment of diabetic cardiomyopathy and concomitant metabolic derangements (Figure 7).

The clinical ramifications of this pathway are substantial, given its ability to reduce body fat mass, restore glucose homeostasis, inhibit the generation of ROS, reduce adipose tissue inflammation, enhance mitochondrial function and most importantly, restore myocardial function. This study demonstrates for the first time that EET upregulation of PGC-1α inhibits NOV, and prevents increased expression of inflammatory markers through upregulation HO-1 in both the heart and pericardial adipose tissue, thwarting the development of cardiomyopathy. Hence, the EET-agonist-mediated reduction of NOV identifies it as a novel pharmacologic target to treat myocardial dysfunction caused by obesity and diabetes mellitus.

## Figures and Tables

**Table 1:** Metabolic parameters in db/db mice

		**Control**	**EET-A**	**EET-APGC1α(sh)**	
**Body Weight (g)**		63.9 ± 1.58	45.2 ± 1.33*	54.9 ± 3.65#	
**Food Intake (g)**		15.2 ± 0.30	14.9 ± 0.59	15.4 ± 0.91	
**Fasting Blood Glucose (mg/dL)**		355 ± 22.2	134 ± 6.5*	205 ± 5.5#	
**Heart Weight (g)**		0.16 ± 0.004	0.13 ± 0.001*	0.14 ± 0.006	
**Blood Pressure (mmHg)**		161.9 ± 4.19	110.3 ± 3.58*	137.9 ± 2.59#	
**VO_2_ (mL/Min)**		25.31 ± 0.501	34.70 ± 7.35*	26.01 ± 13.01#	
**RQ (CO_2_ Eliminated/O_2_ Consumed)**		0.81 ± 0.005	0.75 ± 0.007*	0.82 ± 0.017#	
**EET levels in Cardiac tissue (pg/µg of protein)**		50.93 ± 9.58	83.33 ± 5.10*	55.36 ± 2.15#	
**Glucose Tolerance Test: Mean ± SEM Blood Glucose levels (mg/dl)**					
**Group**	**0**	**30**	**60**	**90**	**120**
**Control**	355.5 ± 19.3	550.0 ± 8.8	600.0 ± 0.0	557.3 ± 13.2	541.8 ± 7.0
**EET-A**	134.0 ± 6.1	316.0 ± 5.7	339.3 ± 10.6	276.0 ± 23.0	237.7 ± 27.6
**EET-APGC-1α (sh)**	205.3 ± 5.2	388.3 ± 40.7	388.0 ± 5.7	372.3 ± 18.7	305.0 ± 4.0
**Lean**	115.0 ± 4.5	318.8 ± 34.6	247.8 ± 16.1	169.3 ± 14.7	135.3 ± 10.7

(Table showing food intake, final body weight, fasting blood glucose as well as glucose tolerance test, heart weight, blood pressure, and oxygen consumption as well as respiratory quotient, and endogenous EET levels in cardiac tissues of control, EET-agonist treated, and EET-agonist treated PGC-1α-deficient db/db mice. Results are mean ± SE, n=6, *p<0.05 *vs.* db/db control, ^#^p<0.05 *vs.* db/db mice treated with EET-agonist alone).
